# Parasitic infection prevalence in tuberculosis patients and their household contacts in the Littoral Region of Cameroon

**DOI:** 10.1016/j.parepi.2025.e00409

**Published:** 2025-01-13

**Authors:** Lucy Cho Nchang, Chefor Magha, Patience Agwa Fonong, Narcisse Victor Tchamatchoua Gandjui, Nancielle Mbiatong Tchatat, Desmond Akumtoh Nkimbeng, Frank Noel Nietcho, Juluis Visnel Foyet, Fanny Fri Fombad, Tatiana Djikeussi Katcho, Jerome Fru Cho, Achim Hoerauf, Manuel Ritter, Samuel Wanji

**Affiliations:** a Parasites and Vector Biology Research Unit (PAVBRU), Department of Microbiology and Parasitology, University of Buea, Buea, Cameroon; b Research Foundation in Tropical Diseases and the Environment (REFOTDE), Buea, Cameroon; c Institute for Medical Microbiology, Immunology and Parasitology (IMMIP), University Hospital Bonn (UKB), Bonn, Germany; d German-West African Centre for Global Health and Pandemic Prevention (G-WAC), Partner Site, Bonn, Germany; e German Center for Infection Research (DZIF), Partner Site Bonn-Cologne, Bonn, Germany

**Keywords:** Parasitic infections, Tuberculosis patients, Household contacts, Associated factors

## Abstract

**Background:**

Parasitic infections are known to suppress the cell mediated immunity that protects against tuberculosis. The status of parasitic infections among bacteriologically confirmed tuberculosis patients and their household contacts in Cameroon is not well established. This study aimed at reporting the status of parasitic infections in TB patients and their household contacts with keen interest in associated risk factors to disease exposure.

**Methodology:**

This was a hospital based cross-sectional descriptive study carried out with newly diagnosed active tuberculosis (TB) patients and their household contacts in the Littoral Region of Cameroon. Socio-demographic data and associated factors were collected using structured questionnaires. Blood, stool, urine and skin snip samples were collected following standard guidelines for investigation of parasitic infections. Descriptive analysis was performed, bivariate analysis was computed and a multivariable analysis was done to provide adjusted odds ratios (AOR).

**Results:**

A total of 712 TB patients and 472 household contacts were recruited. The overall prevalence of parasitic infections in TB patients was 25.84 % (184/712) and household contacts was 31.36 % (148/472). Blood protozoan (*Plasmodium falciparum)* infection among active TB patients (20.22 %) and their household contacts (26.27 %) was the most frequently detected parasitic infection. *Loa loa* was the predominant helminth species seen among active TB patients while *Schistosoma mansoni* was the predominant helminth infection detected in household contacts. TB patients and household contacts living in urban areas had lower odds of being associated with helminth infections (AOR 0.2, 95 % CI: 0.10–0.40; p ˂ 0.0001 and AOR 0.11, 95 % CI: 0.04–0.27; p ˂ 0.0001 respectively) as compared to those residing in rural areas.

**Conclusion:**

We observed that 31 % of the TB patients and household contacts are infected with parasites including P. falciparum, Loa loa and Since helminths can downregulate immune responses against bacterial infections and thus affect treatment efficacy, we recommend that diagnosis of parasitic infections should be included during TB diagnosis and treatment programmes, especially in rural areas.

## 1 Background

Tuberculosis (TB) is a communicable disease caused by the bacillus *Mycobacterium tuberculosis* and is one of the leading causes of death worldwide (WHO, 2022). The bacterium is transmitted by inhalation of viable tubercle bacilli via infectious droplet nuclei, generated by coughing, sneezing, speaking or singing of persons with active tuberculosis (Zumla et al., 2015; CDC, 2019). An estimated global total of 10.6 million people fell ill with TB in 2021, equivalent to 134 cases per 100,000 population, with 6.7 % being among people living with Human Immuno-deficiency Virus (HIV). Current reports revealed an increase in the estimated number of deaths from TB globally between 2019 and 2021 (WHO, 2022).

Humans are host to nearly 300 species of parasitic worms and over 70 species of protozoa, with over a billion people affected by helminth infections causing inflammation (WHO, 2020). A wide range of parasitic infections have been reported to affect humans, especially in developing countries (Wong et al., 2020). Protozoa and helminth parasites are known to affect the gastrointestinal cavity. Intestinal parasites have been reported to affect about one-sixth of the world's population (Harhay et al., 2010) with *Schistosoma mansoni*, *Trichuris trichuria* (whipworm), *Ascaris lumbricoides* (roundworm), *Necator americanus* and *Ancylostoma duodenale* (hookworms) being the most common (Jourdan et al., 2018; *WHO Soil Transmitted Helminth Infections*, 2022).

Africa carries the highest burden of infectious diseases, with a particularly large burden of helminth and protozoan infections (Orish, 2015). These parasitic infections are preventable neglected tropical diseases (Hotez, 2011). People of all ages are at risk of being infected by intestinal protozoa, however, children aged 5–17 years are the most vulnerable (Alemu et al., 2019). Helminth infections are acquired via ingesting eggs or larvae from contaminated water, soil or food or through active skin penetration by infective hookworm larvae in contaminated soil (Jourdan et al., 2018). The majority of the effects of parasites on the organism result in suppression of the immune system of the host, which may contribute to its susceptibility to other diseases (Schmid-Hempel, 2009).

Malaria and intestinal parasites are endemic in Cameroon and are still a cause of morbidity and mortality (Eyong et al., 2021; Mekachie Sandie et al., 2021). Malaria is caused by *Plasmodium* parasites (Fecchio et al., 2020). Intestinal parasites are protozoans and helminths that cause gastrointestinal tract infections (Mekachie Sandie et al., 2021; Wale and Gedefaw, 2022). Filarial worms are nematodes that dwell in the subcutaneous tissues and the lymphatics (Metenou et al., 2012). Nine filarial species infect humans including *Wuchereria bancrofti, Brugia malayi, Onchocerca volvulus, Brugia timori, Loa loa, Mansonella streptocerca, Mansonella perstans, Mansonella ozzardi, and Mansonella sp “DEUX” (*Diemert*, 2012**;* Mourembou et al., *2015**)*. An estimated 200 million persons are infected by filarial parasites worldwide (Metenou et al., 2012). These parasites are transmitted by specific species of mosquitoes or other arthropods and have a complex life cycle including infective larval stages harboured by insects and adult worms that reside in lymphatic or subcutaneous tissues of humans. The offspring of adults are microfilariae that either circulate in the blood or migrate through the skin and are the major antigenic reservoir in the host (Metenou et al., 2012).

Tuberculosis and parasitic infections can cause significant harm in humans (Li and Zhou, 2013; Tigabu et al., 2019) and mostly affect people of low socioeconomic living standards in disadvantaged communities (Teixeira et al., 2019). Climatic conditions, socio-demographic characteristics, and living standards of the population are believed to determine the type of helminths existing in various TB endemic areas (*Nigeria. J. Appl. Sci. Environ. Manag.*, 2022). One of the risk factors to develop TB is the presence of intestinal parasites (Elias et al., 2006; Neto et al., 2009). An enhanced Th1 immune response is important to protect against TB that includes the cytokines interferon-γ (IFN-γ), interleukin 12 (IL12), tumour necrosis factor-α (TNF-α) and Th17 cytokines (IL-17, IL-21, IL-22 and IL-23) (Abate et al., 2015). Reduction in these Th1 cytokines and enhanced Th2 immune responses characterized by IL-4, IL-5, IL-9, IL-10, IL-13, and increased levels of circulating immunoglobulin E (IgE) are associated with bacteria susceptibility (Abate et al., 2015). Indeed, helminths downregulate Th1 and pro-inflammatory and promote Th2 and regulatory immune responses to support their survival in the host (Allen and Maizels, 2011; Ritter et al., 2018). This modulation of the host immunity may have significant consequences for TB reactivation (Kamal and El Sayed, 2006; Babu and Nutman, 2016; Elias et al., 2001; Bewket et al., 2022) and might impair TB-specific immune responses and treatment efficacy. This dynamic would have significant consequences for disease progression and potential conversion from latent to active TB (Babu et al., 2009a; George et al., 2014; Babu et al., 2009b). In addition, it has been shown that *Schistosoma mansoni* might increase host susceptibility to TB by attenuating Th1-type responses and reducing the protective efficacy of the Bacille Calmette-Guerin (BCG) vaccine (Elias et al., 2005; Elias et al., 2008). This supports why our study included BCG-vaccinated children 5–18 years of age. In general, the status of parasitic infections in TB patients and their household contacts in Cameroon is scarce in the literature. This knowledge will therefore inform prioritization and integrated health interventions in health systems within this area.

## 2 Methods

### 2.1 Ethical consideration

An ethical clearance was obtained from the National Ethics Committee for Human Research, Yaoundé (CNERSH) N° 2020/12/1328/CE/CNERSH/SP and renewed (CNERSH) N°2022/10/1499/CE/CNERSH/SP in 2022. Administrative authorization was obtained for all TB centers. The participants were informed about the study and approval was obtained from TB patients and their household contacts by a signed informed consent and or assent form. TB patients diagnosed with parasites were freely treated according to national treatment guidelines. The field team is composed of a medical doctor, state registered nurses, social scientists, laboratory technicians, PhD and master students from the University of Buea. The field members were all trained with the study protocol and data collection instrument through workshops before the start of data collection.

### 2.2 Study design

This was a hospital-based cross-sectional descriptive study carried out with newly diagnosed active TB patients of 5 years and above, as well as BCG-vaccinated household contacts 5–18 years of age. Demographic information, BCG scar, deworming, mass drug administration, as well as previous TB infection history were collected using structured questionnaires. Blood, stool, urine and skin snip samples were collected for investigation of parasitic infections.

### 2.3 Study site

**Fig. 1:**
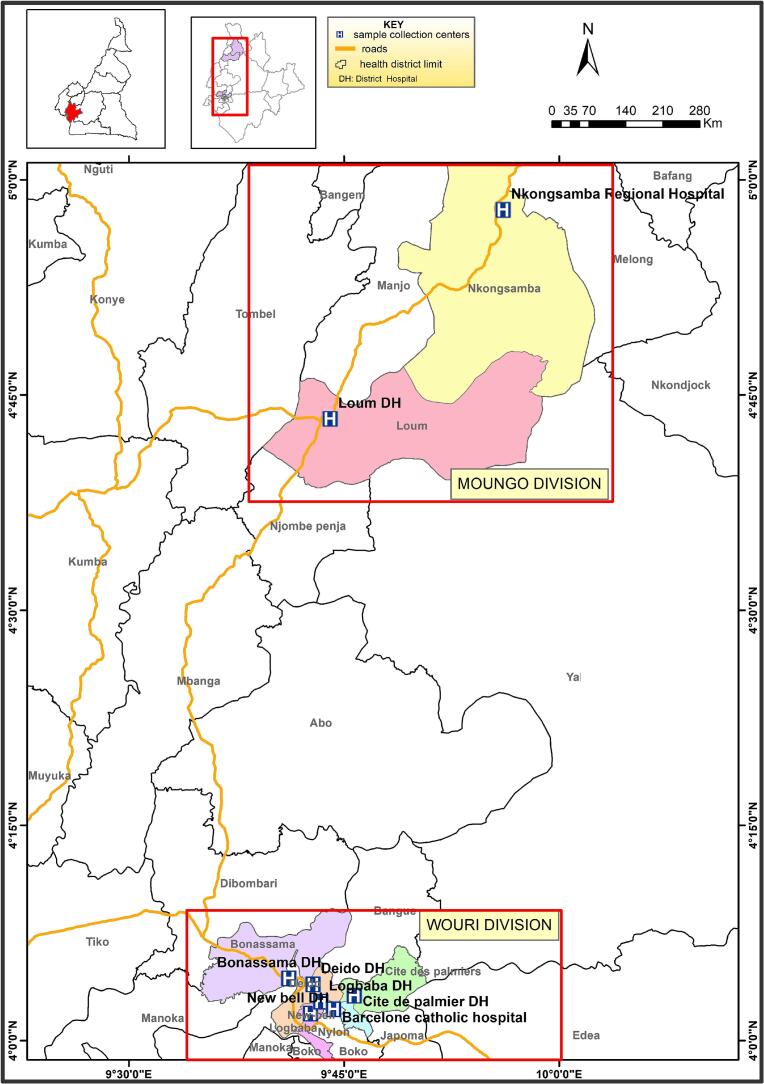
Map showing the eight TB diagnostic and treatment centers selected in the Littoral Region of Cameroon.

The Littoral region in Cameroon has an estimated population of 3,7 million (Population Stat, 2020). This region has a mean altitude of 383 m above sea level. Eight TB diagnostic and treatment centers located in the Moungo and Wouri divisions of the Littoral region in Cameroon were selected including the Nkongsamba Regional Hospital and Loum District Hospital located in the Moungo division, Bonassama District Hospital, Deido District Hospital, NewBell District Hospital, Logbaba District Hospital, Cite des Palmiers District Hospital and Barcelone Catholic Hospital all located in Douala town in the Wouri division (Fig. 1). Douala is a large city, the capital of the Wouri Division and the economic capital of Cameroon.

### 2.4 Study population, selection criteria and recruitment

Adults strictly greater than 5 years, individuals of either sex, newly diagnosed, bacteriologically confirmed pulmonary tuberculosis patients and children between 5 and 18 years of age living with active TB patients were invited to participate in the study. BCG-vaccinated individuals living in the same household as the active TB index patients aged 5–18 years enrolled into the study are referred to as household contacts. Individuals unable or unwilling to provide informed consent, blood/sputum/stool/urine samples for laboratory testing and individuals previously treated or already on anti-TB therapy were excluded. Pregnant women, severely anaemic, disoriented, in severe distress, unconscious or mentally incapacitated individuals, or those with bleeding tendencies as well as unvaccinated children were not included. The field teams worked closely with the TB focal persons to recruit participants from all the centers. Two field teams were involved for the 8 centers in the two divisions. The field team working in Wouri established a focal working point in Barcelone Catholic Hospital, which is located in Douala with well-established infrastructure and state-of-the-art TB diagnostic. Patients that were interested in participating in the study were transferred to this center. The objectives of the study were explained by social scientists in the formal languages (French and English) and were appropriate to the local languages of the participants. TB patients and their household contacts who met the study inclusion criteria were enrolled and participants were recruited from October 2020 to June 2022.

### 2.5 Sample collection and processing

#### 2.5.1 Tuberculosis diagnosis

Tuberculosis diagnosis and treatment in Cameroon is the responsibility of the National TB Control Programme under the Ministry of Public Health. Individuals with symptoms suggestive of TB (such as cough, fever, night sweats and weight loss) are sent for laboratory diagnosis. Investigations are carried out according to national guidelines (PNLT, 2019) using sputum smear microscopy, Xpert MTB/RIF assay or the TB-LAMP assay to establish a bacteriologically confirmed diagnosis of TB. Patients with confirmed TB are registered for anti-TB treatment and monitored with the standard 6-month regimen (2 months of rifampicin/ isoniazid/ pyrazinamide and ethambutol followed by 4 months of rifampicin and isoniazid) (PNLT, 2019). Samples were collected from all participants before commencement of anti-TB therapy. HIV infected individuals were directed to specialized clinics for treatment and further medical support.

#### 2.5.2 Parasite diagnosis

##### 2.5.2.1 Blood

Using 75 μL capillary tubes, thick blood films for filarial detection were prepared using standardized 50 μL capillary blood. Thick blood smears were dehaemoglobinised using tap water for 10 min and were fixed with absolute methanol for 1 min. Smears were later stained in 10 % Giemsa for 45 min, rinsed in distilled water, and air-dried. Slides were read under a light microscope at a magnification of 10×. *Loa loa* microfilaria (mf) were differentiated from *M. perstans* mf by their large size since both filaria are found in peripheral blood. Mf counts were expressed as microfilariae per millilitre of blood, this procedure was performed as described by Wanji et al. (Wanji et al., 2016). Thick and thin blood films were also prepared and stained with a 10 % Giemsa stain solution for diagnosis of *Plasmodium* parasites as earlier described (Sumbele et al., 2020). The malaria parasitaemia was determined under the 1000× magnification of a light microscope. A smear was only considered negative if no malaria parasites were seen after scanning the entire stained smear. For positive smears, the parasite intensity was estimated by counting the parasites against at least 200 leucocytes and parasite density was recorded as the number of parasites/μL of blood while assuming an average leucocyte count of 8000 cells/ μL of blood (Cheesbrough, 2009).

##### 2.5.2.2 Urine

Urine samples (50 mL) were collected in 50 mL sterile falcon tubes, and centrifuged at 1000*g* for 10 min, the supernatant was discarded and the entire sediment was observed microscopically for the presence of *Schistosoma haematobium*. The number of eggs seen was reported per 50 mL of urine (Cheesbrough, 2009).

##### 2.5.2.3 Stool

Stool samples collected in sterile cups were used for detection of gastrointestinal infections by Kato-Katz. For this technique, 41.7 mg of the sieved feces sample was placed on a glass slide using a template. The preparation was then covered with a piece of cellophane soaked in glycerol. Subsequently, the slide was inverted and gently pressed down for a thin smear. The eggs were then identified and counted under the microscope using 10× magnification and expressed in per gram of feces. This technique was performed as previously described (Katz et al., 1972; Vlaminck et al., 2018).

##### 2.5.2.4 Skin

Skin snipping was performed as previously described (Wanji et al., 2015). After clinical examination, two skin biopsies from the posterior iliac crest were taken using a 2 mm corneo-scleral punch (CT 016 Everhards 2218–15C, Germany). The skin samples from each participant were placed in two separate wells of a microtitre plate containing 2 drops of sterile normal saline, the collected skin samples were incubated at room temperature for 24 h. All emerged mf were counted using 10× magnification of a binocular light microscope (Human) and expressed per skin snip, as described by Wanji et al. (Wanji et al., 2015).

### 2.6 Data processing

The data were entered into Microsoft Excel 2016, checked for completeness and analyzed using R software version 4.3.1 (R Core Team, 2023). Binomial logistic regression (with the link function *logit*) was used. Risk factors were studied using both bivariate and multivariate analysis. In the bivariate analysis, the outcome variable was parasitic infection status (1 = positive and 0 = negative) and single predictor variables were included in the model. On the other hand, in the multivariate analysis, all the potential determinants of the parasitic infection were included in the model at once as predictor variables, with the outcome variable being the same as in the bivariate analysis. Crude and adjusted odds ratios, as well as their 95 % confidence intervals, were deduced from bivariate and multivariate models respectively to measure the risk of exposure to parasitic infections. The statistically significant difference was set at p ˂ 0.05. Results were presented using frequencies (percentages) in tables.

## 3 Results

### 3.1 Socio-demographic characteristics of study population

A total of 1184 participants made up of 712 active TB patients and 472 household contacts were recruited into this study. From the 712 active TB patients, 444 (62.36 %) were male and 268 (37.64 %) females. The distribution of participants according to their age groups, residence, and occupation are shown in Table 1. Most participants (73 %) lived in urban areas. The majority of these participants were artisan and students (19.8 % and 19.5 %, respectively). More than 2/3 of the total active TB patients were HIV negative (85.7 %).

**Table 1:** Socio-demographic Characteristics of TB Patient and their Household Contact.

		TB Patients		Household Contacts	
Variable	Category	Frequency	Percent (%)	Frequency	Percent (%)
Sex	Female	268	37.64	247	52.33
	Male	444	62.36	225	47.67
Age group	5–15	27	3.79	–	–
	16–30	299	41.99	–	–
	31–45	254	35.67	–	–
	46–55	63	8.85	–	–
	56–65	46	6.46	–	–
	>65	23	3.23	–	–
Age group	5–9	–	–	191	40.47
	10–14	–	–	197	41.74
	15–18	–	–	84	17.80
Residency	Rural	192	26.97	154	32.63
	Urban	520	73.03	318	67.37
Occupation	Artisan	141	19.80	–	–
	Student	139	19.52	–	–
	salaried	91	12.78	–	–
	Business	83	11.66	–	–
	Farmer	59	8.29	–	–
	Transporter	50	7.02	–	–
	Others	149	20.93	–	–
HIV Status	Negative	610	85.67	–	–
	Positive	91	12.78	–	–
	Absent	11	1.54	–	–
Size of household	2–5	–	–	122	25.85
	6–10	–	–	262	55.51
	11+	–	–	83	17.58
	Absent	–	–	5	1.06

Out of the 472 household contacts of active TB patients included in the study, 247 (52.33 %) were female and 245 (47.67 %) males. Majority of these household contacts (67.37 %) lived in urban areas. Their age group distribution, gender, residence and household size are shown in Table 2.

**Table 2:** Prevalence of Parasitic Infections in TB Patients and Their Household Contacts.

		TB Patients		Household Contacts	
Category	Parasite species	Number of infected individuals	Prevalence (95 % CI)	Number of infected individuals	Prevalence (95 % CI)
Blood Protozoa	*Plasmodium* sp.	144	20.22 (17.43–23.34)	124	26.27 (22.49–30.43)
Helminths	*Loa loa*	15	2.11 (1.27–3.47)	2	0.42 (0.11–1.68)
	*Onchocerca volvulus*	12	1.69 (0.96–2.95)	3	0.64 (0.20–1.95)
	*Schistosoma mansoni*	9	1.26 (0.66–2.41)	8	1.69 (0.85–3.35)
	*Ascaris lumbricoides*	6	0.84 (0.38–1.86)	7	1.48 (0.71–3.08)
	*Taenia* sp.	4	0.56 (0.21–1.49)	0	0
	*S. haematobium*	3	0.42 (0.14–1.30)	7	1.48 (0.71–3.08)
	*Trichuris trichiura*	3	0.42 (0.14–1.30)	4	0.85 (0.32–2.24)
	Hookworms	2	0.28 (0.07–1.12)	0	0
	*Paragonimus* sp.	2	0.28 (0.07–1.12)	0	0
	*Mansonella perstans*	1	0.14 (0.02–0.99)	0	0
	*Dipylidium caninum*	1	0.14 (0.02–0.99)	0	0
	*Dicrocoelium dendriticum*	1	0.14 (0.02–0.99)	1	0.21 (0.03–1.49)
	*Enterobius vermicularis*	1	0.14 (0.02–0.99)	0	0
	*Strongyloides stercoralis*	1	0.14 (0.02–0.99)	0	0
	Overall*	54	7.58 (5.85–9.78)	31	6.57 (4.65–9.20)
Stool protozoa	*Entamoeba histolytica*	4	0.56 (0.21–1.49)	4	0.85 (0.32–2.24)
	*Blastocystis hominis*	2	0.28 (0.07–1.12)	1	0.21 (0.03–1.49)
	*Giardia* spp.	0	0	2	0.42 (0.11–1.68)
	Overall*	6	(0.38–1.87)	7	1.48 (0.71–3.09)

### 3.2 Overall prevalence of parasitic infections in TB patients and their household contacts

Parasitic infections were grouped as follows: blood protozoa, helminths and stool protozoan infections. The overall parasitic infection rate among household contacts (31.36 %) was significantly higher (*p* = 0.040) when compared to active TB patients (25.84 %). A total of 17 species of parasites were detected in active TB patients including one blood protozoa, two stool protozoa and fourteen helminth species. However, in their household contacts, 11 species of parasites were detected (one blood protozoa, three stool protozoa and seven helminth species). Generally, household contacts were significantly more likely to be infected by parasites (OR = 0.76; 95 % CI = 0.59–0.99, *p* = 0.040) compared to active TB patients as shown in Table 3. The prevalence of blood protozoan infection was also significantly lower in active TB patients compared to their household contacts (*p* = 0.035). This was however not the case with helminth infections wherein active TB patients were seen to be more infected by helminth species (7.44 %) as compared to household contacts (5.51 %), even though the difference was not statistically significant.

**Table 3:** Overall prevalence in TB patients and their household contacts based on infection group.

Parasite group	Participant	Number of infected individuals	OR (95 % CI)	*p*-value
Blood Protozoa	Household contacts	124 (26.27 %)	–	–
	TB Patients	144 (20.22 %)	0.74 (0.57–0.98)	**0.035**
Stool Protozoa	Household contacts	7 (1.48 %)	–	**–**
	TB Patients	6 (0.84 %)	0.56 (0.18 1.7)	0.307
Helminths	House contacts	31 (6.57 %)	–	
	TB patients	54 (7.58 %)	1.17 (0.74–1.86)	0.507
Parasites	Household contacts	148 (31.36 %)	–	
	TB patients	184 (25.84 %)	0.76 (0.59–0.99)	**0.040**

### 3.3 General prevalence of parasitic infections in TB patients

The parasite with the highest prevalence (20.22 %) recorded among active TB patients in the study area was *Plasmodium falciparum*. The prevalence of all parasitic infections diagnosed in TB patients is shown in Table 2. *Loa loa* was more frequently detected than all other helminth infections.

A total of 15 active TB patients were infected with *Loa loa*, indicative of a 2.11 % prevalence, and 12 TB patients were infected with *Onchocerca volvulus* indicative of a 1.69 % prevalence. Only one participant in the entire study population (0.14 %) was infected with *Mansonella perstans*. The third most prevalent helminth infection was *Schistosoma mansoni*, 1.26 % (95 % CI: 0.66–2.41) followed by *Ascaris lumbricoides*, 0.84 % (95 % CI: 0.38–1.86) *Taenia* spp., 0.56 % (95 % CI: 0.21–1.49) *Schistosoma haematobium*, 0.42 % (95 % CI: 0.14–1.30) and *Trichuris trichiura*, 0.42 % (95 % CI: 0.14–1.30), respectively. Hookworms, *Paragonimus* spp., *D. caninum*, *D. dendriticum*, *E. vermicularis*, and *S. stercoralis* were also detected in active TB patients with however very low prevalence (Table 4). *Entamoeba histolytica* 0.56 % (95 % CI: 0.21–1.49) and *Blastocyst hominis* 0.28 % (95 % CI: 0.07–1.12) were the two stool protozoa identified among TB patients.

**Table 4:** Factors associated with *Plasmodium falciparum* infection in active TB patients.

Predictor variables	Number of infected individuals	Bivariate analysis		Multivariate analysis	
		OR	p-value	AOR	p-value
Residency			0.12		**0.041**
Rural	32 (17.20 %)	–	–	–	–
Urban	112 (22.49 %)	1.4 (0.91–2.18)	0.13	1.72 (1.03–2.98)	**0.046**
Sex			0.98		0.80
Female	54 (21.09 %)	–	–	–	–
Male	90 (21.03 %)	1 (0.68–1.46)	>0.9	0.89 (0.54–1.46)	0.75
Age group			**0.006**		0.09
5–15	13 (48.15 %)	–	–	–	–
16–30	68 (23.53 %)	0.33 (0.15–0.75)	**0.007**	0.43 (0.13–1.47)	0.16
31–45	39 (16.12 %)	0.21 (0.09–0.48)	**<0.001**	0.24 (0.67–0.89)	**0.028**
46–55	11 (17.74 %)	0.23 (0.08–0.62)	**0.004**	0.35 (0.09–1.49)	0.15
56–65	7 (16.28 %)	0.21 (0.07–0.62)	**0.006**	0.19 (0.04–0.92)	**0.039**
>65	6 (28.57 %)	0.43 (0.12–1.41)	0.2	0.67 (0.11–3.90)	0.60
Occupation			0.29		0.23
Artisan	36 (26.28 %)	–	–	–	–
Business	13 (16.05 %)	0.54 (0.26–1.06)	0.083	0.76 (0.35–1.57)	0.45
Farmer	12 (20.69 %)	0.73 (0.34–1.50)	0.4	0.89 (0.34–2.13)	0.80
Others	32 (23.02 %)	0.84 (0.48–1.45)	0.5	0.69 (0.36–1.32)	0.27
salaried	13 (15.29 %)	0.51 (0.24–1.00)	0.058	0.38 (0.15–0.85)	**0.028**
Student	31 (22.96 %)	0.84 (0.48–1.45)	0.5	0.33 (0.15–0.69)	**0.046**
Transporter	7 (14.29 %)	0.47 (0.18–1.08)	0.093	0.46 (0.17–1.11)	0.10
HIV			0.23		**0.030**
Negative	130 (22.07 %)	–	–	–	–
Positive	13 (15.12 %)	0.63 (0.32–1.13)	0.14	0.44 (0.17–1.01)	0.07
Deworming intake			0.59		0.25
No	89 (20.41 %)	–	–	–	–
Yes	55 (22.18 %)	1.11 (0.76–1.62)	0.6	1.15 (0.05–9.91)	0.5
Deworming taken			0.61		0.49
Albendazole	33 (19.53 %)	–	–	–	–
Mebendazole	14 (25.93 %)	1.44 (0.69–2.92)	0.3	1.43 (0.62–3.20)	0.39
Others	97 (21.04 %)	1.1 (0.71–1.73)	0.7	1.64 (0.55–4.58)	0.35
Positivity			0.83		0.81
Scanty	14 (24.56 %)	–	–	–	–
Grade 1+	53 (2.82 %)	0.87 (0.45–1.75)	0.7	0.81 (0.39–1.73)	0.63
Grade 2+	35 (18.72 %)	0.73 (0.37–1.52)	0.4	0.73 (0.34–1.60)	0.58
Grade 3+	15 (2.65)	0.81 (0.35–1.87)	0.6	0.73 (0.29–1.79)	0.42

Scanty = 1–9 Acid-fast bacillus (AFB) in 1 length, Grade 1+ = 10–99 AFB in 1 length, Grade 2+ = 1–10 AFB per field in at least 50 visual fields, Grade 3+ = More than 10 AFB per field in at least 20 visual fields.

*Plasmodium falciparum* was the parasite with the highest prevalence (26.27 %) recorded among household contacts of active TB patients, followed by *Schistosoma mansoni* (1.69 %), the equal prevalence of *Ascaris lumbricoides* and *Schistosoma haematobium* (1.48 %), *Trichuris trichiura* (0.85 %), *Loa loa* (0.42 %), and *Dicrocoelium dendriticum* (0.21 %), respectively. Stool protozoan infections were the least detected among household contacts (Table 5). *Entamoeba spp* (0.85 %), *Giardia* spp. (0.42 %) and *Blastocyst hominis* (0.21 %) were the three stool protozoa identified among household contacts.

**Table 5:** Risk factors associated with *Plasmodium falciparum* infection in household contacts.

Predictor variables	Number of infected individuals	Bivariate analysis		Multivariate analysis	
		**OR**	**p-value**	**AOR**	**p-value**
Sex			0.732		0.670
Female	63 (25.51)	–	–	–	–
Male	61 (27.11)	1.07 (0.71–1.62)	0.731	1.02 (0.67–1.57)	0.919
Age group			0.583		0.561
5–9	46 (24.08)	–	–	–	–
10–14	53 (26.90)	1.15 (0.73–1.82)	0.544	1.1 (0.69–1.77)	0.689
15–18	25 (29.76)	1.35 (0.75–2.39)	0.307	1.48 (0.81–2.69)	0.196
Household size			**0.039**		**0.038**
02–05	29 (23.8)	–	–	–	–
06–10	62 (23.7)	1.00 (0.61–1.67)	0.997	1.04 (0.62–1.76)	0.880
11+	31 (37.3)	1.95 (1.06–3.61)	**0.032**	2.35 (1.25–4.45)	**0.008**
Residency			0.716		0.979
Rural	39 (25.32)	–	–	–	–
Urban	85 (26.73)	1.09 (0.70–1.70)	0.716	1.02 (0.64–1.65)	0.922
Deworming intake			0.141		0.058
No	71 (23.99)	–	–	–	–
Yes	53 (30.11)	1.37 (0.90–2.08)	0.140	2.21 (0.71–6.39)	0.149
Deworming taken			0.089		0.105
Albendazole	39 (33.33)	–	–	–	–
Mebendazole	8 (18.60)	0.45 (0.18–1.02)	0.069	0.42 (0.16–0.99)	0.057
Others	6 (40.00)	1.32 (0.42–3.92)	0.068	1.21 (0.37–3.63)	0.742

### 3.4 Risk factors associated with *plasmodium falciparum* infection in TB patients and their household contacts

Multivariable analysis for residency, sex, age group, occupation, HIV status, deworming intake and sputum smear positivity for acid-fast bacillus (AFB) were analyzed as factors that could be associated with the presence of *P. falciparum* infection in active TB patients as shown in Table 4. The residency and HIV infection status were significantly associated with *P. falciparium* infection in TB patients. TB patients in urban areas were more likely to be infected with *P. falciparium* than those living in rural areas (AOR 1.72, 95 % CI: 1.03–2.98; *p* = 0.046). TB patients with HIV positive status were less likely to be infected with *P. falciparium* than those with HIV-negative status (AOR 0.44, 95 % CI: 0.17–1.01; *p* = 0.030). Age was not significantly associated with the presence of *P. falciparium* in TB patients even though TB patients within the age ranges 31–45 (AOR 0.24, 95 % CI: 0.67–0.89; *P* = 0.028) and 56–65 (AOR 0.19, 95 % CI: 0.04–0.92; *P* = 0.039) were less likely to be associated with *P. falcipaium* infection than those in the age range 5–15. The occupation of TB patients was not significantly associated with *P. falciparium* infection even though salaried workers and students were less likely to be associated with *P. falciparium* infection than artisans. Factors like sex, sputum smear positivity and de-worming intake were not significantly associated with *P. falciparium* infection in TB patients.

Household contacts with more than ten members were more likely to be infected with *Plasmodium falciparum* infection (AOR 2.35, 95 % CI: 1.25–4.45; *p* = 0.008) compared to those who were between two to five members in the home. Neither residency, sex, age group, occupation nor deworming intake were seen to be associated with *P. falciparum* infection in the household contacts of active TB patients as shown in Table 5.

### 3.5 Factors associated with helminth infections in TB patients and household contacts

The residency of active TB patients and their household contacts was found to be the only factor in this study that was significantly associated with helminth infections. TB patients and their Household contacts living in urban areas were less likely to be associated with helminth infections as compared to those residing in rural areas (AOR 0.38, 95 % CI: 0.19–0.76; *p* = 0.006) and (AOR 0.11, 95 % CI: 0.04–0.27; p ˂ 0.0001) respectively. Sex, age, occupation, HIV status, deworming intake and sputum smear positivity were not significantly associated with helminth infections (Table 6, Table 7).

**Table 6:** Associated risk factors to helminth infections in active TB patients.

Predictor variables	Number of infected individuals	Bivariate analysis		Multivariate analysis	
		OR	p-value	AOR	p-value
Sex			0.199		0.08
Female	16 (6.0)	–	–	–	–
Male	38 (8.6)	1.47 (0.82–2.77)	0.209	1.76 (0.82–4.06)	0.16
Age group			0.536		0.554
5–15	3 (11.1)	–	–	–	–
16–30	21 (7.0)	0.6 (0.19–2.69)	0.440	0.59 (0.14–3.01)	0.477
31–45	15 (5.9)	0.5 (0.15–2.28)	0.302	0.36 (0.08–2.10)	0.219
46–55	7 (11.1)	1 (0.25–4.94)	1.000	0.41 (0.07–2.62)	0.318
56–65	5 (10.9)	0.98 (0.22–5.09)	0.975	0.37 (0.06–2.53)	0.287
>65	3 (13.0)	1.2 (0.20–7.12)	0.834	0.31 (0.04–2.44)	0.254
Residency			**˂ 0.0001**		**˂ 0.001**
Rural	33 (17.2)	–	–	–	–
Urban	21 (4.0)	0.2 (0.11–0.36)	**˂ 0.0001**	0.38 (0.19–0.76)	**0.006**
Occupation			**0.0033**		0.07
Artisan	8 (5.7)	–	–	–	–
Business	2 (2.4)	0.41 (0.06–1.69)	0.267	0.42 (0.06–1.80)	0.29
Farmer	13 (22.0)	4.7 (1.86–12.6)	**0.001**	2.24 (0.73–6.79)	0.15
Student	8 (5.8)	1.02 (0.36–2.84)	0.976	0.84 (0.21–2.58)	0.79
Transporter	2 (4.0)	0.69 (0.10–2.88)	0.650	0.12 (0.01–1.11)	0.12
Salaried	7 (7.7)	1.39 (0.47–4.00)	0.543	1.84(0.21–2.86)	0.79
Others	14 (9.4)	1.72 (0.71–4.44)	0.236	1.62 (0.64–4.28)	0.31
Deworming intake			0.440		0.82
No	37 (8.2)	–	–	–	–
Yes	17 (6.6)	0.79 (0.43–1.42)	0.446	0.89 (0.04–5.14)	0.91
Deworming taken			0.391		0.48
Albendazole	10 (5.6)	–	–	–	–
Mebendazole	6 (10.9)	2.04 (0.67–5.79)	0.186	2.01 (0.57–7.15)	0.24
Others	38 (7.9)	1.44 (0.73–3.11)	0.324	1.34 (0.07–9.22)	0.77
HIV status			0.664		0.24
Negative	48 (7.9)	–	–	–	–
Positive	6 (6.6)	0.83 (0.31–1.85)	0.671	0.46 (0.07–1.75)	0.32
Positivity			0.83		0.73
Scanty	5 (8.77 %)	–	–	–	–
Grade 1+	16 (6.45 %)	0.83 (0.30–2.64)	0.72	0.73 (0.24–2.47)	0.58
Grade 2+	14 (7.49 %)	0.96 (0.35–3.09)	0.94	0.95 (0.31–3.24)	0.92
Grade 3+	8 (10.67)	1.50 (0.47–5.28)	0.49	1.46 (0.41–5.61)	0.56

**Table 7:** Associated risk factors to helminth infections in household contacts of TB patients.

Predictor variables	Number of infected individuals	Bivariate analysis		Multivariate analysis	
		OR	p-value	AOR	p-value
Sex			0.230		0.215
Female	13 (5.3)	–	–	–	–
Male	18 (8.0)	1.57 (0.75–3.34)	0.234	1.6 (0.73–3.58)	0.244
Age group			0.965		0.982
5–9	13 (6.8)	–	–	–	–
10–14	13 (6.6)	0.97 (0.43–2.16)	0.935	1.02 (0.44–2.41)	0.957
15–18	5 (6.0)	0.87 (0.27–2.38)	0.792	0.8 (0.24–2.37)	0.705
Household size			0.144		0.113
02–05	8 (6.6)	–	–	–	–
06–10	21 (8.0)	1.24 (0.55–3.06)	0.615	0.95 (0.40–2.46)	0.914
11+	2 (2.4)	0.35 (0.05–1.45)	0.194	0.54 (0.08–2.47)	0.470
Residency			**˂ 0.0001**		**˂ 0.0001**
Rural	25 (16.2)	–	–	–	–
Urban	6 (1.9)	0.1 (0.04–0.23)	**˂ 0.0001**	0.11 (0.04–0.27)	**˂ 0.0001**
Deworming intake			0.829		0.470
No	20 (6.8)	–	–	–	–
Yes	11 (6.2)	0.92 (0.42–1.94)	0.830	0 (0.00 – Inf.)	0.995
Deworming taken			**0.027**		0.082
Albendazole	11 (9.4)	–	–	–	–
Mebendazole	0 (0.0)	0 (0.00 – Inf.)	0.987	0 (0.00 – Inf.)	0.991
Others	20 (6.4)	0.66 (0.31–1.47)	0.289	0 (0.00 – Inf.)	0.995

### 3.6 Factors associated to stool protozoan infections in TB patients and their household contacts

Despite apparent differences observed in the prevalence of stool protozoan infections concerning modalities of predictor variables, none was found to be significantly associated in active TB patients as well as their household contacts. Details are shown in supplements S1 and S2.

Generally, our findings reveal more household contacts infected with parasites compared to active TB patients. Specifically, active TB patients were more infected with helminths than household contacts and only one participant was diagnosed with *M. perstans* infection. Residence was an important factor associated with helminth infections, where both active TB patients and household contacts living in rural areas were more likely to be exposed to helminth infections when compared to those in urban areas.

## 4 Discussion

The overall prevalence of parasitic infections in active TB patients and their household contacts in the Littoral region of Cameroon was found to be 25.84 % and 31.36 %, respectively. These findings are lower than a similar study carried out in Cameroon where a prevalence of 46 % was reported among TB patients (Weldeslassie et al., 2023). All participants positive for malaria had *Plasmodium falciparum*, which is not surprising because Cameroon has been known to be an endemic zone for this particular species (Wanji et al., 2003). In this study, blood protozoan (*Plasmodium falciparum)* infection (20.22 %) among TB patients and their household contacts (26.27 %) was the most frequently detected. These results are in line with a study carried out in Cameroon where 28.0 % malaria prevalence was reported among TB patients (Weldeslassie et al., 2023). This finding is however lower than a study carried out in Angola, in which a prevalence of 37.5 % was reported (Valadas et al., 2013) and higher than that of other studies done in a hospital-based setting in Cameroon at 1.5 % (Irene et al., 2016), Uganda at 2.2 % (Baluku et al., 2019) as well as Tanzania at 4.3 % (Range et al., 2007a). This was possibly due to the differences in sample sizes, endemicity and seasonal/climatic factors of various study sites which were not within the scope of indicators this study evaluated.

The findings of this study show a 7.58 % prevalence of helminths and 0.84 % prevalence for intestinal protozoa in TB patients. However, the prevalence of these infections was higher than reports from other studies carried out in Ethiopia (5.8 %) (Tesfaye et al., 2022), and lower than reports from rural China (14.9 %) (Li et al., 2014), systematic review and meta-analysis in Iran (26 %) (Taghipour et al., 2019), India (27.11 %) (Panigrahi et al., 2019), and Tanzania (29.5 %) (Mhimbira et al., 2017). Additionally, household contacts in our study had 6.57 % prevalence of helminths and 1.48 % prevalence of stool protozoan infections. Higher prevalence of intestinal helminths has also been reported in Ethiopia with 71 % among TB patients and 36 % in their household contacts (Elias et al., 2006), including intestinal parasites prevalence of 22 % in TB patients and 9 % in household contacts (Alemu et al., 2019). The low prevalence rates seen in this study could be attributed to personal hygiene, the differences in the diagnostic techniques used to detect parasites, geographical differences and socio-economic factors.

A wide range of parasitic infections were detected in TB patients and their household contacts in this study with however very low prevalence rates. Our study found *Schistosoma mansoni* infection as the predominant helminth infection among household contacts (1.69 %) and the third most prevalent helminth infection in active TB patients (1.26 %). In the Littoral region of Cameroon, Loum locality has been highlighted as an endemic zone for schistosomiasis (Tchuem Tchuenté et al., 2003; Nkengni et al., 2019). The prevalence of *Ascaris lumbricoides* (0.84 %) in active TB patients was higher than reports from Tanzania (0.6 %) (Mhimbira et al., 2017) and lower than the prevalence reported from TB patients in Cameroon (1.5 %) (Weldeslassie et al., 2023), Ethiopia (1.2 %) (Tesfaye et al., 2022) and meta-analysis in Iran (6 %) (Taghipour et al., 2019). This study found 2.11 % loiasis, 1.69 % onchocerciasis, and 0.14 % mansonellosis prevalence in active TB patients. One study in Cameroon reported a lower prevalence (1 %) of loiasis (Weldeslassie et al., 2023). Lower prevalence rates were seen for *Onchocerca volvulus* and *Loa loa* (0.64 % and 0.42 %) respectively in household contacts. The least helminth parasites observed in the TB patients (0.14 %) were *Mansonella perstans*, *Dipylidium caninum*, *Dicrocoelium dendriticum*, *Enterobius vermicularis* and *Strongyloides stercoralis*. However, a systematic review reported *S. mansoni*, *S. stercoralis* and hookworm to be among the leading helminths in TB patients in Africa. The lower prevalence of *S. stercoralis* and *Enterobius vermicularis* in our study could be due to differences in diagnostic techniques. There is no standard diagnostic method for *S. stercoralis* (World Health Organization, 2020) even PCR was shown to have a lower sensitivity in the diagnosis of light-intensity *S. stercoralis* infection (Knopp et al., 2014). However, recent findings reported higher sensitivity with Baermann and sedimentation/concentration methods (Nieves et al., 2024). Since these techniques were not applied in this study, likely, *Strongyloides* infections were not properly detected. Similar, the most reliable method for *E. vermicularis* diagnosis is the cellophane tape test or pinworm paddle test (Jardine et al., 2006). *Dipylidium caninum* and *Dicrocoelium dendriticum* are the causes of zoonotic diseases that rarely affect humans (Cabeza-Barrera et al., 2011; Rousseau et al., 2022). This could account for the low prevalence seen in this study. In addition, this study might be among the first to report these zoonotic parasites in TB patients. With regards to *M. perstans* the reason for this very low prevalence among active TB patients could be because, tuberculosis is a disease which is highly prevalent in cities and crowded areas, while mansonellosis is mostly seen in rural areas where helminths are more prevalent. The difference in distribution could account for the low prevalence.

Generally, TB patients were less likely to be infected with parasites than the household contacts (OR = 0.76, 95 % CI: 0.59–0.99; *p* = 0.040). In this study, the multivariate analysis showed that residency and HIV status of the TB patients were significantly associated with the presence of *P. falciparium* infection. Logistic regression analysis showed that the odds of having *P. falciparium* infection among active TB patients who lived in urban areas significantly increased by a factor of 1.72 compared to those in rural areas. There are currently no related studies that have reported residency as a predictor of *P. falciparium* in active TB patients. However, the general trend indicates that the odds of having malaria is higher in those living in rural areas than in urban areas (Alemu et al., 2011; Wilson et al., 2015). There is a growing concern about the risk of urban malaria. WHO framework for the response to the rise of urban malaria predicts that, unplanned rise in urbanization will likely increase the burden of malaria, especially in the urban poor (World Health Organization, 2022). Furthermore, TB patients with HIV infection were less likely to be infected with *Plasmodium falciparium* than HIV negative patients. However, another study indicated that the presence of malaria in TB patients was a predictor of having HIV infection (Range et al., 2007b). Nonetheless, with regards to household contacts there was an association between household size and *Plasmodium falciparium* infection (*P* = 0.008). The odds of contracting *Plasmodium falciparum* infection among household contacts who lived in homes with more than ten members significantly increased by a factor of 2.35 when compared to those in homes of two to five members. These findings are in line with reports from India, where families of 4–5 members and six or more members had considerable higher chances of having malaria cases as compared with families of ≤3 members (Sharma et al., 2015).

Multivariate analysis also indicated that residency was significantly associated with helminth infections in TB patients and their household contacts. TB patients and their household contacts living in urban areas had lower odds of having helminth infections (AOR 0.2, 95 % CI: 0.10–0.41; p ˂ 0.0001 and AOR 0.11, 95 % CI: 0.04–0.27; p ˂ 0.0001, respectively) compared to those residing in rural areas. A similar finding in Ethiopia indicates that TB patients residing in rural areas were significantly associated with parasitic infections (Tesfaye et al., 2022). Similar findings have been reported in Ethiopia with presumptive TB patients living in urban areas were less likely to be associated with intestinal parasitic infections than those living in rural areas (Belete et al., 2024).

In general, an important strength of this study is the large sample size and the fact that both TB patients and their household contacts were included. However, there were also a few limitations. This was a purely descriptive study and hence no conclusions could be made concerning the clinical outcome of parasitic infections in active TB patients and household contacts. Additionally, household contacts were not tested for latent or active tuberculosis unless they showed signs of a TB infection. The household contacts were also not in the same age group as TB patients, hence could not be seen as the corresponding healthy control group. Further studies need to include age-matched contacts to pinpoint risk factors for TB, especially to reveal parasitic infections that might promote TB.

## 5 Conclusions

This study has shown that TB patients and BCG-vaccinated household contacts are infected with parasitic infections. The most identified parasites in TB patients were *Plasmodium falciparium*, *Loa loa*, *Onchocerca volvulus* and *Schistosoma mansoni* and the least parasites were *Mansonella perstans*, *Dipylidium caninum*, *Dicrocoelium dendriticum*, *Enterobius vermicularis*, *Strongyloides stercoralis*, *Entamoeba histolytica* and *Blastocystis hominis*. In BCG-vaccinated household contacts, *P. falciparum*, *S. mansoni*, *Ascaris lumbricoides* and *S. haematobium* were mostly seen. The least parasites were *Loa loa*, *Dicrocoelium dendriticum*, *Entamoeba spp*, *Giardia* spp. and *Blastocystis hominis*. HIV status and residency were significantly associated with the presence of *P. falciparium* infection in TB patients while the household size was significantly associated with the presence of *P. falciparium* infection in household contacts. Residency was significantly associated with the presence of helminth infections in TB patients and their household contacts. We therefore recommend that more immunological and clinical studies should be carried out to understand the dynamic between HIV and *P. falciparium* infection in TB patients. Secondly, malaria control programme should not neglect malaria control efforts in urban areas especially in TB patients. Lastly, the diagnosis of parasitic infections needs to be implemented during the TB control and treatment programmes to minimize risk factors for disease progression and treatment failure.

## Consent for publication

Not applicable.

## Funding

This work was funded by the 10.13039/501100001659Deutsche Forschungsgemeinschaft (DFG) within the “African-German Cooperation Projects in Infectiology” (HO 2009/14–1). AH is additionally supported by the DFG under Germany's Excellence Strategy – EXC2151–390873048 and SW is the Senior Fellow Plus of the European and Developing Countries Clinical Trials Partnership (EDCTP2).

## CRediT authorship contribution statement

**Lucy Cho Nchang:** Writing – review & editing, Writing – original draft, Methodology, Formal analysis. **Chefor Magha:** Writing – review & editing, Writing – original draft, Methodology, Formal analysis. **Patience Agwa Fonong:** Writing – review & editing, Methodology. **Narcisse Victor Tchamatchoua Gandjui:** Writing – review & editing, Methodology, Formal analysis. **Nancielle Mbiatong Tchatat:** Writing – review & editing, Methodology. **Desmond Akumtoh Nkimbeng:** Writing – review & editing, Methodology. **Frank Noel Nietcho:** Writing – review & editing, Methodology. **Juluis Visnel Foyet:** Writing – review & editing, Methodology. **Fanny Fri Fombad:** Writing – review & editing, Methodology. **Tatiana Djikeussi Katcho:** Writing – review & editing, Methodology. **Jerome Fru Cho:** Writing – review & editing, Methodology. **Achim Hoerauf:** Writing – review & editing, Validation, Supervision, Resources, Project administration, Funding acquisition, Conceptualization. **Manuel Ritter:** Writing – review & editing, Writing – original draft, Validation, Supervision, Project administration, Methodology, Investigation, Funding acquisition, Formal analysis, Conceptualization. **Samuel Wanji:** Writing – review & editing, Writing – original draft, Validation, Supervision, Resources, Project administration, Methodology, Investigation, Funding acquisition, Formal analysis, Conceptualization.

## Declaration of competing interest

The authors declare that they have no competing financial interests.

## Data Availability

The data sets used in this study are available upon request from the corresponding author.

## Appendix A Supplementary data

Risk factors for stool protozoan in active TB patients and their household contacts Image 1
