# Supplementary material for: Parasitic infection prevalence in tuberculosis patients and their household contacts in the Littoral Region of Cameroon
Source: Parasite Epidemiol Control. 2025 Jan 13;28:e00409. doi: 10.1016/j.parepi.2025.e00409 (PMC7617333; doi:10.1016/j.parepi.2025.e00409)
Supplement: Supplementary file 1 — Risk factors for stool protozoan in active TB patients and their household contacts [file mmc1.docx]

Supplementary Table S1: Risk factors to stool protozoan infections in active TB patients

| **Predictor variables** | **Number of infected**  **individuals** | **Bivariate analysis** | | **Multivariate analysis** | |
| --- | --- | --- | --- | --- | --- |
|  |  | **OR (95% CI)** | **p-value** | **AOR (95% CI)** | **p-value** |
| Residency |  |  | 0.73 |  | 0.73 |
| Rural | 2 (1.04%) | — | — | — | — |
| Urban | 4 (0.77%) | 0.74 (0.14 - 5.34) | 0.7 | 1.39 (0.19 - 15.0) | 0.8 |
| Sex |  |  | 0.54 |  | 0.54 |
| Female | 3 (1.12%) | — | — | — | — |
| Male | 3 (0.68%) | 0.6 (0.11 - 3.27) | 0.5 | 0.82 (0.13 - 5.16) | 0.8 |
| Age group |  |  | 0.69 |  | 0.73 |
| 5 - 15 | 1 (3.70%) | — | — | — | — |
| 16 - 30 | 2 (0.67%) | 0.18 (0.02 - 3.84) | 0.2 | 0.08 (0.00 - 2.48) | 0.11 |
| 31 - 45 | 2 (0.79%) | 0.21 (0.02 - 4.53) | 0.2 | 0.03 (0.00 - 1.23) | 0.05 |
| 46 - 55 | 1 (1.59%) | 0.42 (0.02 - 10.9) | 0.5 | 0.05 (0.00 - 2.91) | 0.14 |
| 56 - 65 | 0 (0.00%) | 0 | >0.9 | 0 | >0.9 |
| >65 | 0 (0.00%) | 0 | >0.9 | 0 | >0.9 |
| Occupation |  |  | 0.52 |  | 0.35 |
| Business | 1 (1.20%) | — | — | — | — |
| Farmer | 1 (1.69%) | 1.41 (0.06 - 36.2) | 0.8 | 3.13 (0.09 - 113) | 0.5 |
| Others | 1 (0.67%) | 0.55 (0.02 - 14.1) | 0.7 | 0.59 (0.02 - 16.9) | 0.7 |
| salaried | 2 (2.20%) | 1.84 (0.17 - 40.1) | 0.6 | 3.01 (0.23 - 75.0) | 0.4 |
| Student | 1 (0.72%) | 0.59 (0.02 - 15.2) | 0.7 | 0.16 (0.00 - 6.79) | 0.3 |
| Transporter | 0 (0.00%) | 0 | >0.9 | 0 | >0.9 |
| Artisan | 0 (0.00%) | 0 | >0.9 | 0 | >0.9 |
| HIV |  |  | 0.4 |  | 0.50 |
| Negative | 4 (0.66%) | — | — | — | — |
| Positive | 2 (2.20%) | 3.4 (0.47 – 17.7) | 0.2 | 3.1 (0.37 - 20.5) | 0.2 |
| Deworming intake |  |  | 0.49 |  | 0.78 |
| No | 3 (0.66%) | — | — | — | — |
| Yes | 3 (1.16%) | 1.76 (0.32 - 9.60) | 0.5 | 0 | >0.9 |
| Deworming taken |  |  | 0.30 |  | 0.41 |
| Albendazole | 3 (1.69%) | — | — | — | — |
| Mebendazole | 0 (0.00%) | 0 | >0.9 | 0 | >0.9 |
| Others | 3 (0.63%) | 0.36 (0.07 - 1.99) | 0.2 | 0 | >0.9 |

Supplementary Table S2: Risk factors to stool protozoan infections in household contacts of active TB patients

| **Predictor variables** | **Number of infected**  **individuals** | **Bivariate analysis** | | **Multivariate analysis** | |
| --- | --- | --- | --- | --- | --- |
|  |  | **OR (95% CI)** | **p-value** | **AOR (95% CI)** | **p-value** |
| Residency |  |  | 0.180 |  | 0.252 |
| Rural | 4 (2.6) | — | — | — | — |
| Urban | 3 (0.9) | 0.36 (0.07 – 1.64) | 0.181 | 0.48 (0.09 – 2.33) | 0.359 |
| Sex |  |  | 0.613 |  | 0.600 |
| Female | 3 (1.2) | — | — | — | — |
| Male | 4 (1.8) | 1.47 (0.32 – 7.54) | 0.615 | 1.45 (0.30 – 7.74) | 0.639 |
| Age group |  |  | 0.236 |  | 0.224 |
| 5 – 9 | 1 (0.5) | — | — | — | — |
| 10 – 14 | 5 (2.5) | 4.95 (0.79 – 95.3) | 0.146 | 5.51 (0.86 – 107) | 0.124 |
| 15 – 18 | 1 (1.2) | 2.29 (0.09 – 58.4) | 0.560 | 2.25 (0.09 – 58.2) | 0.571 |
| Household size |  |  | 0.247 |  | 0.189 |
| 02 – 05 | 2 (1.6) | — | — | — | — |
| 06 – 10 | 5 (1.9) | 1.17 (0.25 – 8.23) | 0.856 | 1.24 (0.25 – 9.02) | 0.805 |
| ≥11 | 0 (0.0) | 0.00 | 0.993 | 0.00 | 0.996 |
| Deworming intake |  |  | 0.761 |  | 0.874 |
| No | 4 (1.4) | — | — | — | — |
| Yes | 3 (1.7) | 1.27 (0.25 – 5.81) | 0.759 | 0.00 | 0.998 |
| Deworming taken |  |  | 0.342 |  | 0.486 |
| Albendazole | 3 (2.6) | — | — | — | — |
| Mebendazole | 0 (0.00) | 0.00 | 0.992 | 0.00 | 0.997 |
| Others | 4 (1.3) | 0.49 (0.11 – 2.54) | 0.360 | 0.00 | 0.998 |
